# Anaesthetic management of a large paraganglioma resection in a woman with isolated L-looped transposition of the great arteries: a case report

**DOI:** 10.1186/s12871-020-00998-9

**Published:** 2020-04-06

**Authors:** Ling Lan, Penghao Liu, Yuan Tian, Bo Zhu, Le Shen, Yuguang Huang

**Affiliations:** Department of Anaesthesiology, Peking Union Medical College Hospital, Peking Union Medical College and Chinese Academy of Medical Sciences, 100730 Beijing, P.R. China

**Keywords:** Paraganglioma, L-transposition of the great arteries, Goal-directed fluid therapy, Transesophageal echocardiography, Case report

## Abstract

**Background:**

Reports of anaesthetic management of paraganglioma resection in patients with isolated L-transposition of the great arteries (L-TGA) are rare. We focus on the preoperative evaluation, intraoperative management, and postoperative care of a frail patient with “physiologically corrected” L-TGA for paraganglioma resection.

**Case presentation:**

We performed general anaesthesia for a 46-year-old patient with “physiologically corrected” L-TGA undergoing open large retroperitoneal paraganglioma resection. Although the preoperative medical therapy had attained its goals, the patient went through three periods of severe episodic hypertension and tachycardia as tumour manipulation released catecholamines. Goal-directed fluid therapy based on pulse pressure variation (PPV) and point-of-care transesophageal echocardiography (TEE) imaging enabled anaesthesiologists to make rapid judgments and to regulate blood pressure in a timely manner, thereby reducing the risk of heart failure caused by massive rapid fluid bolus therapy. The patient was transferred to the intensive care unit because of intraoperative hemodynamic changes and significant blood loss. Despite transient myocardial injury (elevated troponin I), no lethal arrhythmia or complications occurred perioperatively, and the patient recovered well and was discharged 1 week later.

**Conclusions:**

Goal-directed fluid therapy combined with the adoption of TEE could effectively guide fluid administration, which is helpful for anaesthesia management during operation. We recommend the routine use of TEE in such cases.

## Background

Pheochromocytoma or paraganglioma (PPGL) is a rare neuroendocrine tumour, with an incidence of 0.6 cases per 100,000 person years [1]. L-transposition of the great arteries (L-TGA) is another rare form of congenital heart disease (CHD) characterized by atrioventricular and ventriculoarterial discordance, with a published incidence ranging from 0.02 to 0.07 per 1000 live births, comprising less than 1% of all CHDs [2]. Resecting a PPGL is a high-risk surgical procedure, especially for patients with heart issues. Additionally, a multidisciplinary team is required to reduce peri-anaesthetic risks and result in good perioperative outcomes [3]. Cardiovascular and hemodynamic variables must be monitored closely. However, there have been no standard guidelines for fluid administration during PPGL surgery. After preoperative vasodilation and adequate volume expansion, intraoperative goal-directed fluid therapy may be recommended for guiding fluid administration. We report a case of the anaesthetic management of a successful resection of a large paraganglioma in a woman with isolated L-TGA. Goal-directed fluid therapy and the application of intraoperative transoesophageal echocardiography (TEE) could effectively assess the patient’s cardiac structure and function and guide fluid administration, which are helpful for anaesthesia management during operation.

## Case presentation

We obtained written informed patient consent for this case report. A 46-year-old woman presented to the hospital with paroxysmal dizziness accompanied by visual blurring. Her vital signs, bilateral carotid ultrasound and cranial CT were normal. Admission electrocardiogram (ECG) showed that Q waves and ST segment elevated greater than 0.1 mv and T wave inversion in the right-sided precordial leads,which indicated an inferior myocardial infarction, whereas the level of troponin I was normal. Fast echocardiography revealed congenital heart disease with “physiologically corrected” L-TGA. The echo showed left atrial and anatomic right ventricular enlargement, moderate tricuspid insufficiency and a small amount of pericardial effusion. The preoperative value of NT-proBNP was 148 pg/ml. Because of “physiologically corrected” L-TGA, she did not have any symptoms of discomfort until she was middle-aged. The paroxysmal dizziness and visual blurring could be caused by orthostatic hypotension, which may reflect a low plasma volume or systemic ventricular dysfunction and systemic atrioventricular valve regurgitation. Fast ultrasound also showed a big solid and cystic tumor in the abdomen. Abdominal CT prescribed by the consultant urologist confirmed an 8.5-cm solid and cystic tumor of the left retroperitoneum with haemorrhagic changes inside, closely related to the left adrenal gland. After contrast enhancement, the tumour was significantly enhanced, and a clearance delay was observed. The plasma fractionated metanephrine test showed that normetanephrine was 7.76 nmol/L and metanephrine was 0.66 nmol/L. Iobenguane I-131 scintigraphy showed high uptake of the tumour. Biochemical and radiological evaluation indicated the diagnosis of benign paraganglioma [4], and the surgery was scheduled 2 weeks later. She had not been treated at other hospitals before and she denied there had been a similar situation in her family. The patient was unmarried, and her parents were both died at their early age because of the so called “heart disease”.

For preoperative treatment [5], doxazosin was given 2~4 mg PO qd for 2 weeks. The preoperative treatment was prolonged because of the patient’s poor compliance. She did not take the medicine and monitor BP on time at home. At the time of surgery, she had been in the hospital for another 1 week and was on phenoxybenzamine 10 mg PO bid. Adrenergic blockade was accompanied by a high-sodium diet (5000 mg per day) and generous fluid intake (2.5 l per day). During preoperative preparation in the hospital, the patient had no symptoms of discomfort, and the hemodynamic variables were stable with a BP of 100~110/70~80 mmHg and an HR of 60~80 bpm.

**Fig. 1:**
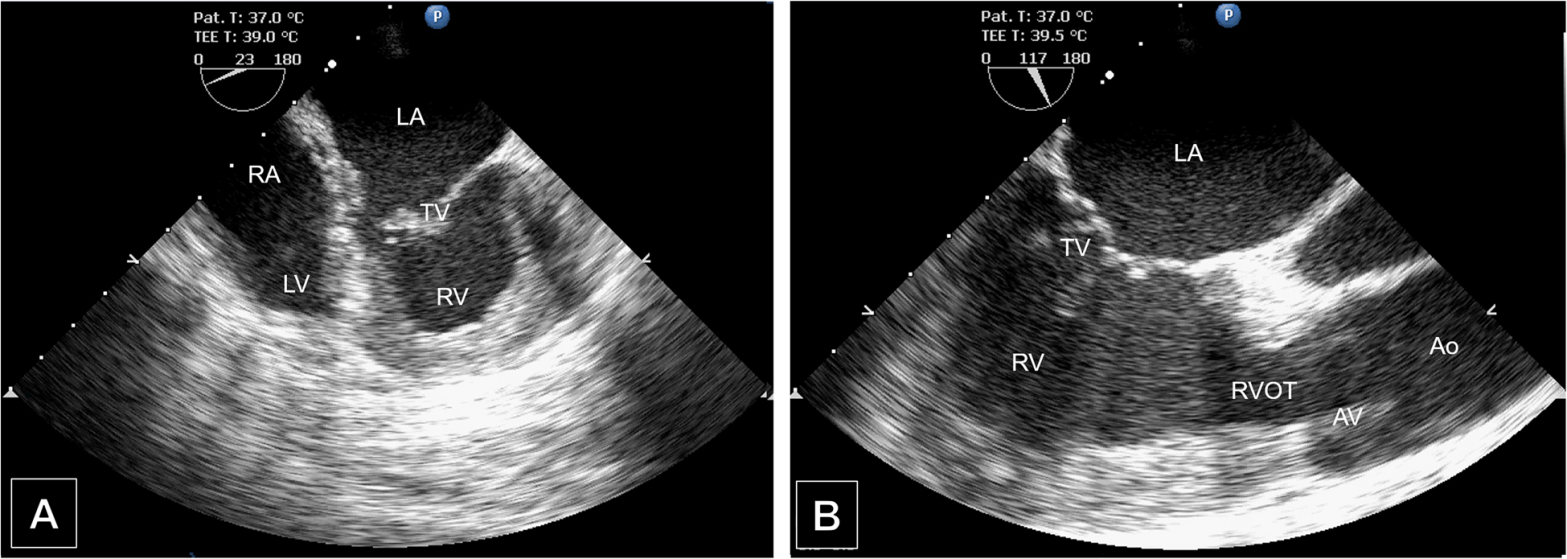
**a** Intraoperative transoesophageal ultrasound of the middle esophageal four-chamber view. The left atrium (LA) was associated with the morphologic right ventricle (RV) through the tricuspid valve (TV), and the right atrium (RA) was associated with the morphologic left ventricle (LV) through the mitral valve. **b** Intraoperative transoesophageal ultrasound of the middle oesophageal aortic valve long-axis view. The aorta (Ao) was connected to the morphologic right ventricle through the aortic valve (AV)

When the patient arrived in the operating room, standard American Society of Anaesthesiologists monitors (e.g., blood pressure, electrocardiography, oxygen saturation) were applied. Her blood pressure (BP) was 108/73 mmHg, her heart rate (HR) was 70 bpm, and her pulse oximeter saturation was 100% while breathing room air. The bispectral index monitor (BIS) was used, and the baseline data were 97. The BIS declined to 86 when midazolam 1 mg iv was administered to allay patient’s anxiety. After a large-bore venous access was placed, the arterial catheter was placed under local anaesthesia prior to induction. Anaesthesia was then induced smoothly with propofol 2 mg/kg iv, lidocaine 1 mg/kg iv, fentanyl 2 μg/kg iv and rocuronium 1 mg/kg iv. Her trachea was intubated, and 8~10 ml/kg tidal volume-controlled ventilation without positive end-expiratory pressure (PEEP) was applied for the patient to an EtCO_2_ of 35~45 mmHg. Anaesthesia was maintained with remifentanil 0.1~0.3 μg/kg/min and 1~1.5% sevoflurane in nitrous oxide/oxygen to keep the BIS between 40 and 60. After the insertion of a right internal jugular central venous line, a TEE probe was placed, and the examination showed that the left atrium was associated with the morphologic right ventricle through the tricuspid valve (Fig. 1a), and the aorta was connected to the morphologic right ventricle (Fig. 1b). An additional movie file shows this in more detail [see Additional file 1].

**Fig. 2:**
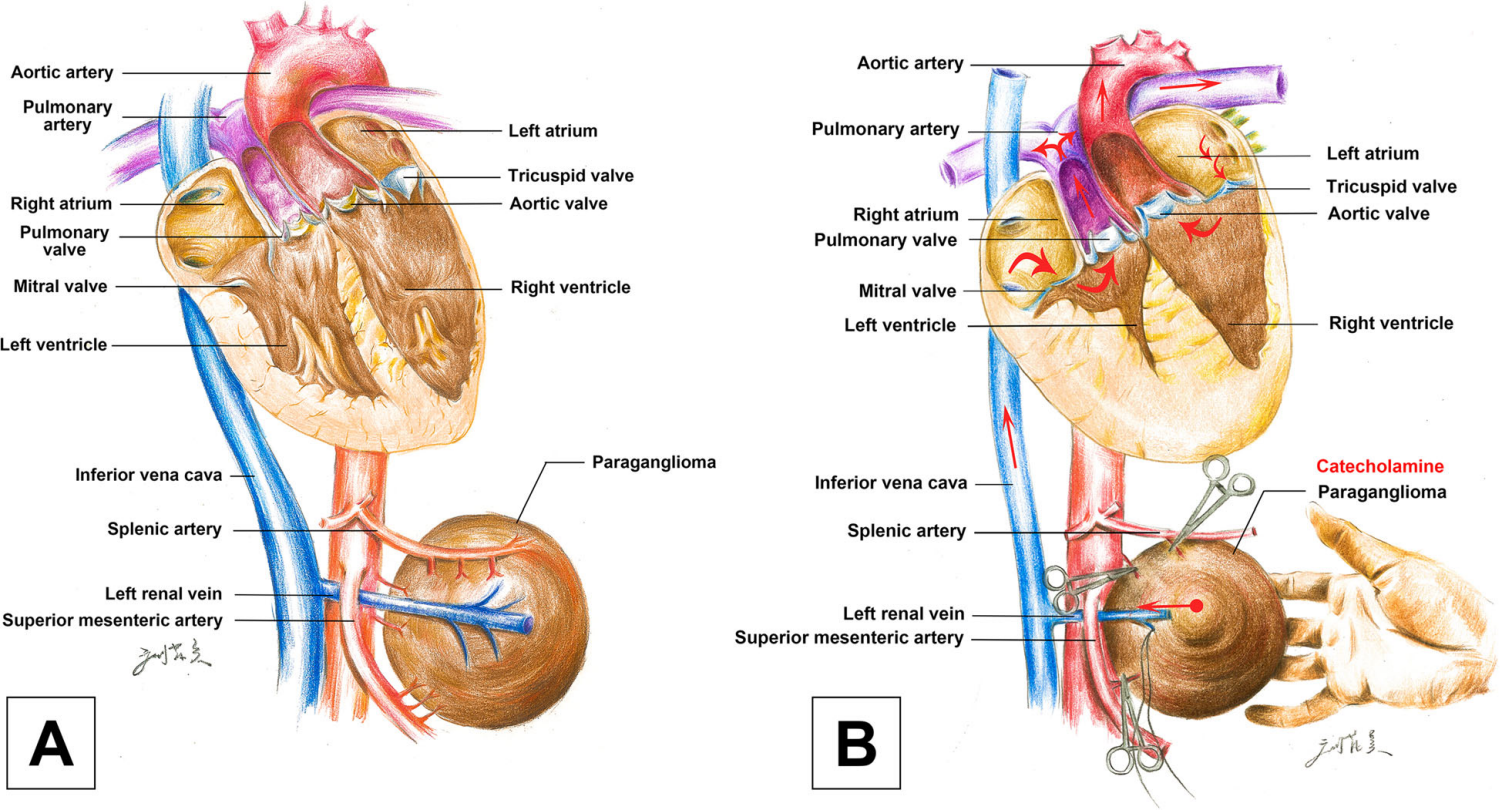
**a** Normal status of the patient without catecholamine release. **b** Status of hemodynamic fluctuation with catecholamine release. Red arrow showing the pattern of blood flow during surgery in this patient

**Fig. 3:**
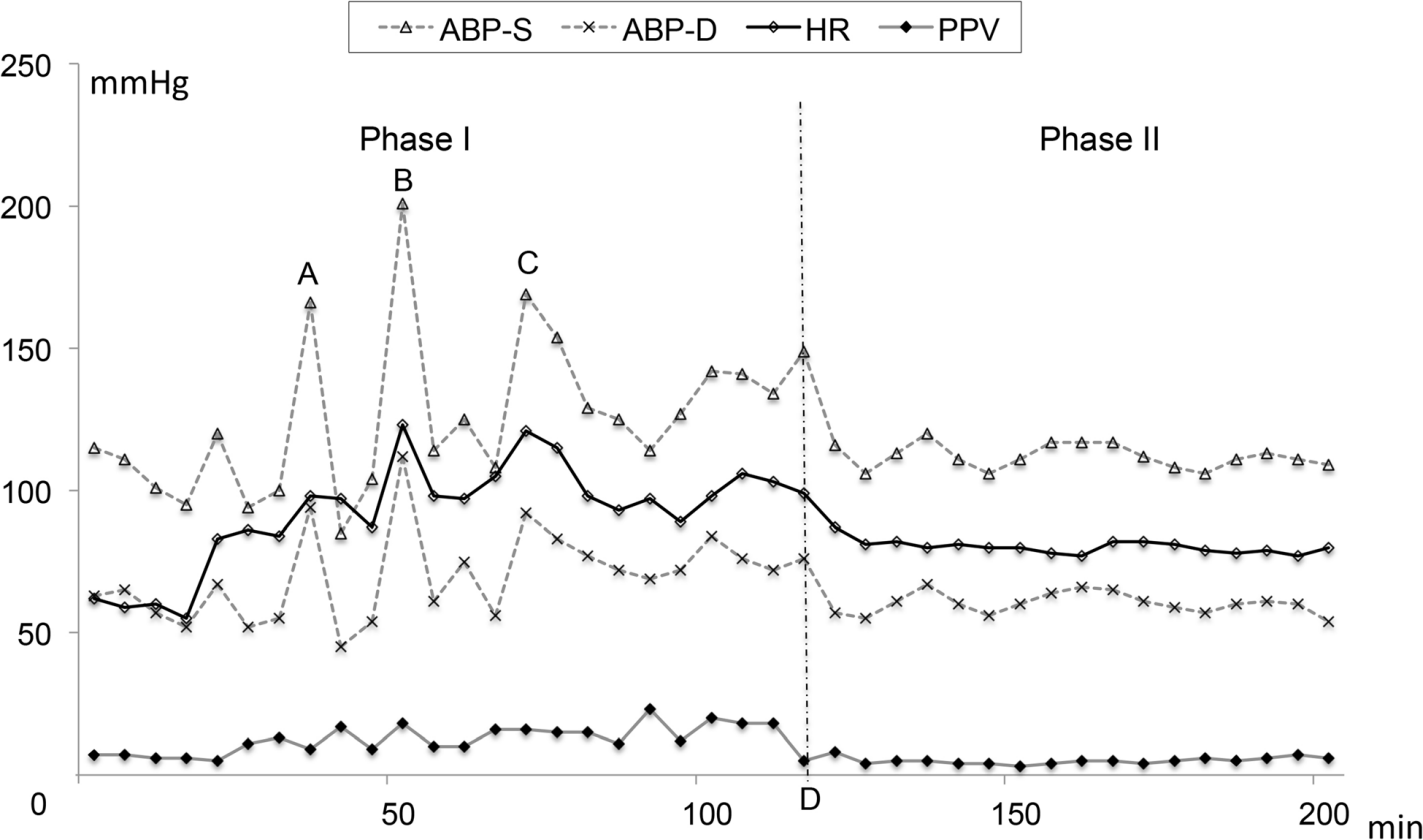
Intraoperative hemodynamic changes in two phases based on the ligation of the blood supply to the tumour. Point **a**, point **b** and point **c** were the three main episodic hypertension instances in Phase I, which occurred before the clamping of the effluent vein. Point **d** was the time when the effluent vein was clamped. Arterial blood pressure-systolic (ABP-S); arterial blood pressure-diastolic (ABP-D); heart rate (HR); pulse pressure variation (PPV)

The operation was performed through an arc incision along the left rib margin. The whole process were divided into two phases based on the ligation of the blood supply to the tumour. Phase I included the portion of surgery during which the tumour was dissected and the vascular supply was isolated before the clamping of the effluent vein. Phase II was the portion of surgery after the effluent vein was clamped. During the induction and initial dissection, the hemodynamic variables were relatively stable, with a mean arterial BP of 65~85 mmHg, HR of 50~80 bpm and PPV of 5%~ 7%. However, BP and HR increased during tumour manipulation because of the release of catecolamine (Fig. 2 simulated the release process of catecholamine). There were mainly three episodes of systolic BP increases to 170~200/100~110 mmHg and HR increases to 110~130 bpm, all lasting approximately 5 mins (Fig. 3). These elevations were treated with continuous infusion of sodium nitroprusside 0.5~3 μg/kg/min and repeated intravenous bolus injections of phentolamine 1 mg and esmolol 10~20 mg. The PPV varied above 15% during the episodic hemodynamic fluctuations. The hypotension was first treated by fluid bolus therapy according to the PPV. TEE was used to provide a reliable and reproducible index of the morphologic RV (the functional LV) end-diastolic volume and ejection fraction. The point-of-care image helped us assess cardiac movement and volume status, especially when hemodynamic fluctuations violently occurred. In addition, TEE demonstrated moderate tricuspid valve regurgitation during the BP increases, but function returned to baseline when BP normalized. According to the real-time TEE image, the intraoperative fluid administration strategy was to mainly maintain reasonable the morphologic RV (the functional LV) filling pressure but to try to limit crystalloid administration to avoid volume overload and acute heart failure. Once the tumour was removed, the sodium nitroprusside infusion was discontinued, and a norepinephrine infusion of 0.05~0.15 μg/kg/min was started to prevent hypotension because the PPV was 3%~ 8%. Intraoperative blood loss was approximately 800 ml, and 4 units of allogeneic red blood cells were infused.

The patient was transferred to the intensive care unit (ICU) because of intraoperative hemodynamic changes and significant blood loss. She was extubated and discharged from the ICU on the first postoperative day. Although the levels of troponin I and NT-proBNP after surgery were both slightly higher than normal, which met the diagnosis of myocardial injury [6], there were no lethal complications. One week later, the patient was discharged home smoothly. During a follow-up call 1 month later, she was very satisfied with the treatment, except for a slight incision pain. Her pathologic testing revealed “cellular changes consistent with paraganglioma” with positive immunostaining for chromogranin A, SDHB and S100.

## Discussion

PPGLs are catecholamine-secreting tumours of chromaffin cells with frequent germline, somatic, or postzygotic mutations in genes. At present, more than 17 pathogenic genes are known to be related to PPGL. Hereditary PPGL accounts for 35%~ 40%, showing familial inheritance, and is one of the manifestations of some hereditary syndromes. Among germline genetic mutations, the highest mutant frequency was in SDHB (10.3%). The PGL4 gene, also known as SDHB, was first identified in 2001 and is also linked to the development of PPGL [7]. Patients with germline SDHB gene mutations less commonly develop multiple tumours. However, the tumours can be distributed in all locations, most commonly in the abdomen. The pathological immunohistochemical staining of SDHB was positive in this patient, which further confirmed the preoperative diagnosis.

According to the different signal transduction pathways involved in the gene mutation of PPGLs, the genes can be mainly divided into two categories. Cluster 1 is related to the hypoxia-related pathway. Cluster 2 promotes tumour growth by activating MAPK and/or mTOR signalling pathways [8]. A common feature of cluster 1 tumours is the activation of hypoxia-inducible factors (HIFs). Patients who had chronic hypoxemia due to cyanotic congenital heart disease were at increased risk for developing PPGL. A population-based retrospective analysis provided direct evidence that patients with CHD had a higher risk of developing PPGL than those with noncyanotic CHD and patients without CHD [9]. PPGL in patients with cyanotic CHD has led to a growing recognition that their cooccurrence is more than coincidence. Vaidya and her colleagues reported that the high frequency (80%) of mutations in EPAS1 in patients with cyanotic CHDs contrasts with rates of only 5 to 6% in cohorts of unselected patients with PPGLs [10]. In addition to chronic hypoxemia, transcription factors are physiologically induced in response to low cellular oxygen levels (hypoxia). Pseudohypoxia occurs when HIF pathways are constitutively activated, regardless of oxygen levels. The patient we reported had isolated L-TGA without any cardiac defects, which was “physiologically corrected” and had no hypoxia because systemic deoxygenated venous blood returns to the pulmonary circulation and oxygenated pulmonary venous blood returns to the systemic circulation [2, 11]. Although the patient did not develop true hypoxemia, cluster 1 genetic mutations could result in the aberrant and constitutive activation of HIFs even under normal levels of oxygen.

The perioperative management of patients with PPGLs has been reviewed in the literature [3, 12–14]. Although no large-scale study has been performed to report perioperative anaesthesia management in patients with PPGLs and CHD, many single-episode cases have been reported about the perioperative management experience in patients with PPGLs and CHD. In particular, cases of cyanotic CHD, including Tetralogy of Fallot and univentricular and Fontan heart disease, have been reported [15, 16]. However, reports of anaesthetic management in patients with isolated L-TGA and paraganglioma are rare.

For preoperative assessment, her BP was normal, and her symptoms were less common for patients with the classic triad of PPGLs, which consists of episodic headache, sweating, and tachycardia [5]. Arrhythmias were another concern because of L-TGA and potential intraoperative catecholamine release. Her preoperative ECG was misinterpreted as an inferior myocardial infarction. These are the characteristic ECG findings of L-TGA because the interventricular septum is depolarized in the opposite direction of normal [17]. In addition, patients with L-TGA are at increased risk for AV heart block and heart failure as adults due to a progressive decline in morphologic right ventricular function. The risk of complete heart block rises over time, with a 2% per year increase in incidence because of progressive fibrosis with advancing age. By 45 years of age, > 30% of patients with isolated L-TGA develop clinical congestive heart failure [18], although there were no signs of catecholamine-induced cardiomyopathy in this patient.

Regarding intraoperative management, many patients who undergo paraganglioma resection exhibit labile BP, arrhythmias, and tachycardia during surgery, though most can be managed without lasting morbidity or mortality [19]. However, our patient was at particular risk from hemodynamic changes and acute morphologic right heart failure. Because of the large tumour size, open adrenalectomy was chosen as recommended by the endocrine society clinical practice guidelines to ensure complete tumour resection and prevent tumour rupture [5]. Open resection could avoid the effects of CO_2_-peritoneum on hemodynamic (catecholamine release, decreased preload, increased afterload, tachycardia, and hypertension). Although adequate filling pressures were necessary in this patient for her tumour resection, intravascular fluid overload was a concern considering her history of subclinical right ventricular dysfunction. A systematic review has demonstrated that a PPV of at least 13 to 15% is strongly associated with volume responsiveness [20]. In this case, goal-directed fluid therapy was adopted, and PPV was used as an indicator of fluid responsiveness. Besides,the point-of-care TEE could be helpful for anaesthesia management during operation. But it requires experienced operators for TEE monitoring.

Postoperative concerns for patients after PPGL resection include recovery of normal adrenergic function with stable BP, potential for rebound hypoglycaemia, and possible adrenal insufficiency. Fortunately, the patient recovered very well despite a brief period of hypotension and myocardial injury. She did not suffer from hypoglycaemia or persistent adrenal dysfunction.

## Conclusions

Goal-directed fluid therapy combined with the adoption of TEE could effectively guide fluid administration, which is helpful for anaesthesia management of paraganglioma resection in patients with isolated L-TGA. We recommend the routine use of TEE in such cases.

## Supplementary information

**Additional file 1.** Loops 1: Middle esophagus four-chamber view. The normal right atrium (RA) is connected to the triangular-shaped ventricle (the morphologic left ventricle) by means of mitral valve with two leaflets at a higher attachment point. The ventricle has small muscle trabeculae. The left atrioventricular valve has the lowest attachment point, suggesting tricuspid valve. The enlarged left atrium (LA) is connected to the round ventricle (the morphologic right ventricle) by the tricuspid valve, which has a thick muscular trabecula. Loops 2: Middle esophagus aortic valve long axis view. The Aorta is closer to the front of the body, and part of the normal riight ventricular structure is missing. The left atrium is enlarged and connected to the morphologic Right Ventricle (the functional left ventricle) by the tricuspid valve.

## Data Availability

All data generated or analysed during this study are included in this published article.

## Abbreviations

L-TGA: L-transposition of the great arteries
PPGL: Pheochromocytoma or paraganglioma
CHD: Congenital heart disease
TEE: Transesophageal echocardiography
PPV: Pulse pressure variation
BP: Blood pressure
HR: Heart rate
ECG: Electrocardiogram
BIS: Bispectrality index
EtCO_2_: Capnography
ICU: Intensive care unit
HIFs: Hypoxia-inducible factors
LA: Left atrium
LV: Left ventricle
RA: Right atrium
RV: Right ventricle
RVOT: Right ventricle outflow tract
AV: Aortic valve
TV: Tricuspid valve
Ao: Aorta
ABP-S: Arterial blood pressure-systolic
ABP-D: Arterial blood pressure-diastolic
